# Combinatory Treatment with miR-7-5p and Drug-Loaded Cubosomes Effectively Impairs Cancer Cells

**DOI:** 10.3390/ijms21145039

**Published:** 2020-07-17

**Authors:** Ewa Gajda, Marlena Godlewska, Zenon Mariak, Ewa Nazaruk, Damian Gawel

**Affiliations:** 1 Department of Biochemistry and Molecular Biology, Centre of Postgraduate Medical Education, Marymoncka 99/103, 01-813 Warsaw, Poland; ewa.gajda@cmkp.edu.pl; 2 Department of Neurosurgery, Medical University of Bialystok, M. Sklodowskiej-Curie 24A, 15-276 Bialystok, Poland; zenon.mariak@umb.edu.pl; 3 Faculty of Chemistry, University of Warsaw, Pasteura 1, 02-093 Warsaw, Poland; enaz@chem.uw.edu.pl; 4 Department of Immunohematology, Centre of Postgraduate Medical Education, Marymoncka 99/103, 01-813 Warsaw, Poland

**Keywords:** miR-7-5p, cubosomes, multidrug resistance, cancer cells, glioblastoma, temozolomide, doxorubicin

## Abstract

Background: Multidrug resistance (MDR) is an emerging problem in the treatment of cancer. Therefore, there is a necessity for novel strategies that would sensitize tumor cells to the administered chemotherapeutics. One of the innovative approaches in fighting drug-resistant tumors is the treatment of cancer with microRNA (miRNA), or the use of cubosomes (lipid nanoparticles) loaded with drugs. Here, we present a study on a novel approach, which combines both tools. Methods: Cubosomes loaded with miR-7-5p and chemotherapeutics were developed. The effects of drug- and miRNA-loaded vehicles on glioma- (A172, T98G), papillary thyroid- (TPC-1) and cervical carcinoma-derived (HeLa) cells were analyzed using molecular biology techniques, including quantitative real-time PCR, MTS-based cell proliferation test, flow cytometry and spheroids formation assay. Results: The obtained data indicate that miR-7-5p increases the sensitivity of the tested cells to the drug, and that nanoparticles loaded with both miRNA and the drug produce a greater anti-tumor effect in comparison to the free drug treatment. It was found that an increased level of apoptosis in the drug/miRNA co-treated cells is accompanied by an alternation in the expression of the genes encoding for key MDR proteins of the ABC family. Conclusions: Overall, co-administration of miR-7-5p with a chemotherapeutic can be considered a promising strategy, leading to reduced MDR and the induction of apoptosis in cancer cells.

## 1. Introduction

Currently, one of the extensively explored strategies in the treatment of cancer is the use of drug-loaded nanoparticles. Numerous studies, including our own previous works, have highlighted the application of drug-loaded lipid-based phases in the successful delivery of chemotherapeutics to cancerous cells in vitro [1,2,3]. Amongst the best-characterized self-assembled lipid liquid crystalline phases are the inverted cubic nanostructures (cubosomes) [4]. Monoolein cubosomes (MO) demonstrate a relatively low cytotoxicity, a high cargo loading capacity and a controlled (prolonged) drug release capability. Cubosomes have been extensively studied because of their ability to maintain their three-dimensional structure under a range of physiologically relevant conditions. The advantage of cubosomes over other lipid nanostructures, e.g., liposomes, lies in the liquid crystalline 3D internal structure, providing a large internal surface area able to efficiently bind large amounts of drugs (both hydrophilic and hydrophobic). The cubosome structure consists of two interpenetrating, non-contacting, aqueous channels, which are surrounded by a lipid bilayer arranged in a thermodynamically favorable periodic 3D structure. Moreover, it has been shown that nanoparticles additionally armed with specific ligands are capable of binding to specific receptors preferably expressed by cancer cells. This allows for the targeting of tumor cells, and increases the efficiency of drug delivery [2]. However, multidrug resistance (MDR), which is frequently developed by aggressive cancer cells, significantly limits the benefits of the controlled and sustained delivery of drugs.

The strategy of using specific microRNAs (miRNAs/miRs) is another alternative and promising tool for treating tumors [5]. The altered expression of miRNAs in various human malignances is well-defined, and their role as potential biomarkers or therapeutic agents has been highlighted. MicroRNA molecules are non-coding RNAs of 19–25 nucleotides, and are involved in the post-transcriptional modulation of gene expression. It has been suggested that they can regulate the expression of 30% of human genes, including other miRNAs [6]. Numerous reports have highlighted the role of miRNAs in the process of cancerogenesis, but intriguingly, it was found that the same miRNA molecules might act as a suppressor and/or an oncogene, depending on the organ or tissue [7,8,9]. Moreover, the significant role of miRNAs in the regulation of genes involved in the process of MDR was emphasized based on high-throughput screening and the use of bioinformatics databases [10,11].

Despite the classic hallmarks of tumor cells, the phenomenon of MDR is another factor that limits the outcome of the treatment of various types of cancer. Therefore, it is necessary to find novel therapeutic strategies. MDR is generally associated with the activity of several membrane proteins. Among 49 ABC transporters linked with the phenomenon of MDR, P-glycoprotein (PGP/*ABCB1*), breast cancer resistance protein (BCRP/*ABCG2*), and multidrug resistance-associated proteins 1 and 6 (MRP1/*ABCC1* and MRP6/*ABCC6*, respectively) are the most studied. It has been shown that their high expression correlates with poor prognosis in patients [12,13,14].

Apart from the ABC family proteins, other mechanisms and proteins are also linked with the MDR phenotype. This includes the activation of DNA repair enzymes, e.g., O^6^-methylguanine methyltransferase (MGMT), which efficiently removes DNA lesions induced by alkylating chemotherapeutics, such as temozolomide (TMZ), leading to failure of the therapy [15]. Drug resistance, caused by high expression of MGMT, is frequently observed in primary glioblastoma (GB) tumors. Multiple studies have reported several miRNAs, including miRNA-370-3p, miRNA-181d, miRNA-767-3p, miRNA-648 and miRNA-603, connected with the modulation of MGMT expression and the restoring of GB cell sensitivity to TMZ [16,17,18,19]. Recent studies by Medarova et al. [11] have demonstrated that therapeutic miRNAs can be identified based on a sequence of nucleotides, which match miRNA candidates to targeted mRNA. Two novel miRNAs (hsa-miR-4261 and hsa-miR-6836-3p), with relevance to multidrug resistance resulting from the activity of the ABC family or MGMT proteins, were designated as potential anti-cancer therapeutics [11]. Nevertheless, there are still no effective methods for improving the clinical outcome of treatment or increasing the patient’s chances of successful therapy.

Here, we present a study on the sensitization of tumor cells to drugs, which engages both strategies: lipid nanocarriers and miRNAs. To our knowledge, the combination of miRNA and drugs loaded into cubosomes has not been reported before. It was assumed that supplementing miRNAs with a chemotherapeutic agent may effectively reverse tumor chemoresistance, and at the same time, sustained drug delivery would efficiently kill cancer cells. The majority of studies focused on GB-derived model cell lines, since GB is the most frequent, aggressive and drug-resistant primary brain tumor found in adults [20,21]. GB is classified as a grade IV cancer, and most new cases are recorded among people aged 50–64 years old, as the risk of developing brain tumors increases with age. Despite therapy, most people die within 15–23 months, with a 5-year survival rate around 6% [21]. We observed that the tested GB tissue samples present altered expression of miR-7-5p, and significantly reduced expressions of PGP, MRP1 and MRP6 encoding genes. Taking this into account, we constructed cubosomes loaded with TMZ or doxorubicin (DOX) and delivered them to cells pre-treated with miR-7-5p, and observed the increased apoptosis of GB cells. Moreover, we enriched this method and increased the effectiveness of treatment by creating cubosomes co-loaded with the drug and miR-7-5p. Overall, the obtained data indicate that miR-7-5p increases the sensitivity of glioma cells to the drug, and that nanoparticles loaded with the chemotherapeutic produce a greater therapeutic effect compared to the usage of the free drug. Finally, it was found that cells exposed to miR-7-5p present reduced expressions of MDR proteins, and therefore increased chemosensitivity. It was also shown that the correlation between miR-7-5p expression and MDR is likely universal, and does not depend on the origin of the cancer cell line or the used drug.

## 2. Results

### 2.1. Downregulation of miR-7-5p in GB Specimens Is Accompanied by an Altered Expression of MDR Genes

**Figure 1:**
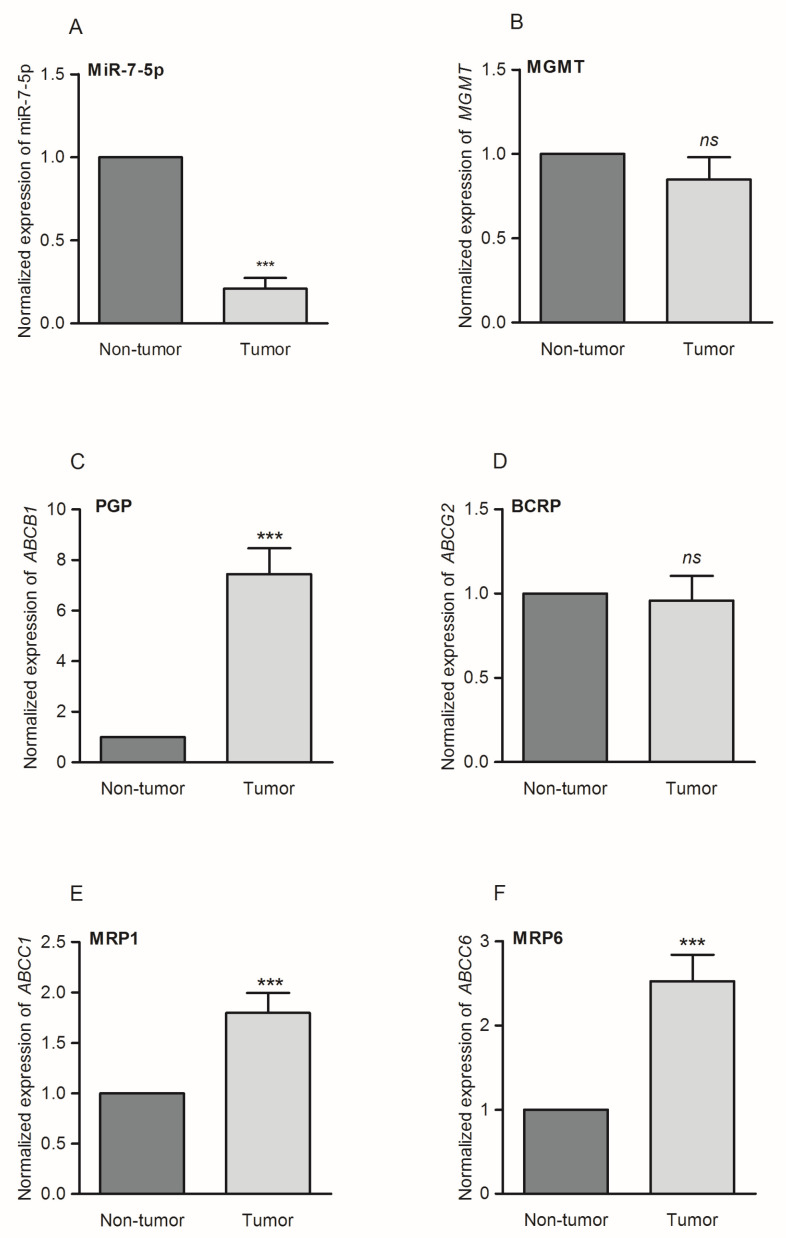
Hsa-miR-7-5p (miR-7-5p) downregulation in glioblastoma (GB) specimens (*n* = 23) is accompanied by alternations in the expression level of genes encoding for multidrug resistance (MDR) proteins. (**A**) Normalized expression of endogenous miR-7-5p; (**B**) normalized expression of the *MGMT* gene encoding for MGMT DNA repair protein; (**C**) normalized expression of the *ABCB1* gene encoding for PGP protein; (**D**) normalized expression of the *ABCG2* gene encoding for BCRP protein; (**E**) normalized expression of the *ABCC1* gene encoding for MRP1 protein; (**F**) normalized expression of the *ABCC6* gene encoding for MRP6 protein. The data, which were obtained using quantitative real-time PCR (RT-qPCR), are presented as mean ± SD. As a non-tumor control, commercial reference FirstChoice Human Brain RNA was used. *** *p* < 0.001; ns, non-significant.

It has been suggested that miR-7-5p may play an important role in oncogenesis [22,23,24,25]. Its expression was reported to be downregulated in GB specimens [26]. As the poor prognosis of GB patients is linked to the frequently observed development of multidrug resistance by tumor cells, we considered that the phenomena of MDR and the depletion of miR-7-5p expression might be related. To test this hypothesis, an expressional panel of major efflux pumps encoding genes, including *ABCB1*, *ABCG2*, *ABCC1* and *ABCC6* (encoding for PGP, BCRP, MRP1 and MRP6 proteins, respectively), was analyzed in a set of 23 GB specimens. In the case of TMZ treatment, an alternative path leads to the development of the MDR phenotype, and therefore the level of *MGMT* expression was established. In GB cells without methylation of the promoter of the *MGMT* gene encoding for the O^6^-methylguanine methyltransferase repair protein, the level of the MGMT enzyme is high, resulting in the removal of TMZ-induced lesions from DNA [27,28,29]. Evaluation of miR-7-5p and MDR genes’ expressions was performed using RT-qPCR. Commercial FirstChoice Human Brain Reference RNA served as a non-tumor control. Firstly, it was confirmed that the expression of miR-7-5p is significantly altered in the tested GB samples (Figure 1A). It was established that deficiency in miR-7-5p did not alter the level of the *MGMT* transcript in the tested specimens (Figure 1B). However, it was found that for four of the screened ABC family genes, a decreased intracellular level of miR-7-5p was associated with the upregulation of three of them. The expression of *ABCB1*, *ABCC1* and *ABCC6* genes (encoding for PGP, MRP1 and MRP6, respectively) was enhanced between 2- and 7-fold (Figure 1C,E,F). The only unaffected MDR gene was *ABCG2*, encoding for the BCRP protein (Figure 1D). A detailed analysis of GB specimens is presented in the supplementary materials (Figure S1).

### 2.2. Increased Intracellular Level of miR-7-5p Results in Enhanced Sensitivity of Tumor Cells to Chemotherapeutics

**Figure 2:**
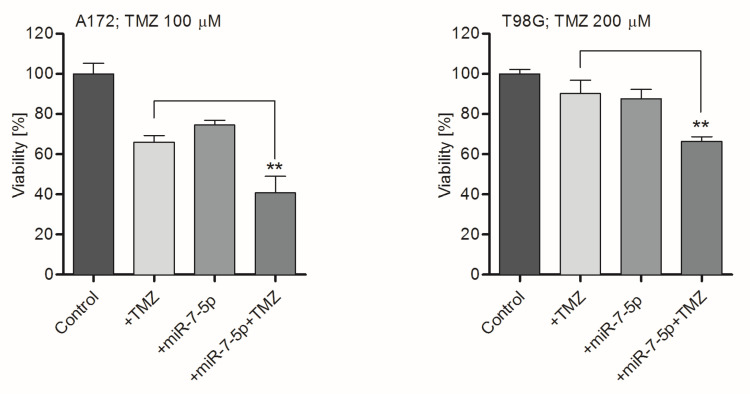
Cells transfected with miR-7-5p present an enhanced sensitivity to temozolomide (TMZ). The viability analysis (MTS assay) of GB-derived A172 and T98G cells treated with miR-7-5p and/or TMZ (100 and 200 µM, respectively) for 48 h. Cells treated with miR-negative (miR-NEG) served as a control. Data are presented as mean ± SD. ** *p* < 0.01.

**Figure 3:**
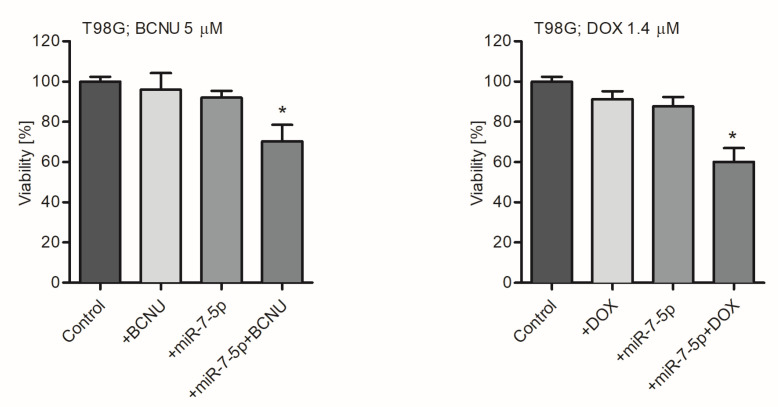
The effect of miR-7-5p is not drug-specific. MiR-7-5p sensitizes drug-resistant T98G cells to carmustine (BCNU) and doxorubicin (DOX). Viability analysis (MTS assay) of the T98G cell line treated with miR-7-5p and BCNU (5 µM; left panel) or DOX (1.4 µM; right panel) for 48 h. Cells treated with miR-NEG served as a control. Data are presented as mean ± SD. * *p* < 0.05.

To further elucidate the link between miR-7-5p depletion and the development of drug resistance in tumor cells, TMZ-sensitive A172 and TMZ-resistant T98G GB-derived cell lines were used in the study. It was found that transfection of A172 cells with miR-7-5p resulted in a significant (~20-fold) increase in the intracellular level of miR-7-5p microRNA (Figure S2), and that transfected cells exhibited a greater sensitivity to chemotherapeutics. As shown in Figure 2, the transfection of cells with miR-7-5p, followed by TMZ treatment, caused a significant decrease in the viability of A172 and T98G cells. In contrast, there was a weak effect on the viability of GB-derived cells when miR-7-5p or TMZ were added independently. As TMZ, an alkaline pro-drug belonging to the class of imidazotetrazinones, is the first choice in the treatment of GB [20,30], we also considered whether the effect of miR-7-5p was limited only to TMZ, or can also be observed when other drug classes are administered. The potential effect of the co-treatment of GB-derived cells with miRNA and alternative drugs was examined using carmustine (BCNU), which is a substitute chemotherapeutic agent used in the treatment of GB [30], and DOX, a standard and commonly used drug in general chemotherapy [31,32]. It was observed that a combinatory treatment of the drug-resistant T98G cell line with miR-7-5p and DOX, when the concentration of the latter is 1.4 µM, sensitizes cells, resulting in a significant (~30%) decrease in their survival rate (Figure 3). Similarly, the effect of the combined treatment was also observed when miR-7-5p was co-administrated with BCNU at the concentrations of 5 and 25 µM (Figure 3). MiRNA-transfected T98G cells were more susceptible (at least five times) to the drug treatment than control cells, in which miR-7-5p was not pre-delivered (Figure S3).

### 2.3. The Effect of miR-7-5p Is Not Cell-Type Specific and Is Associated with a Decrease of Expression in MDR Encoding Genes

**Figure 4:**
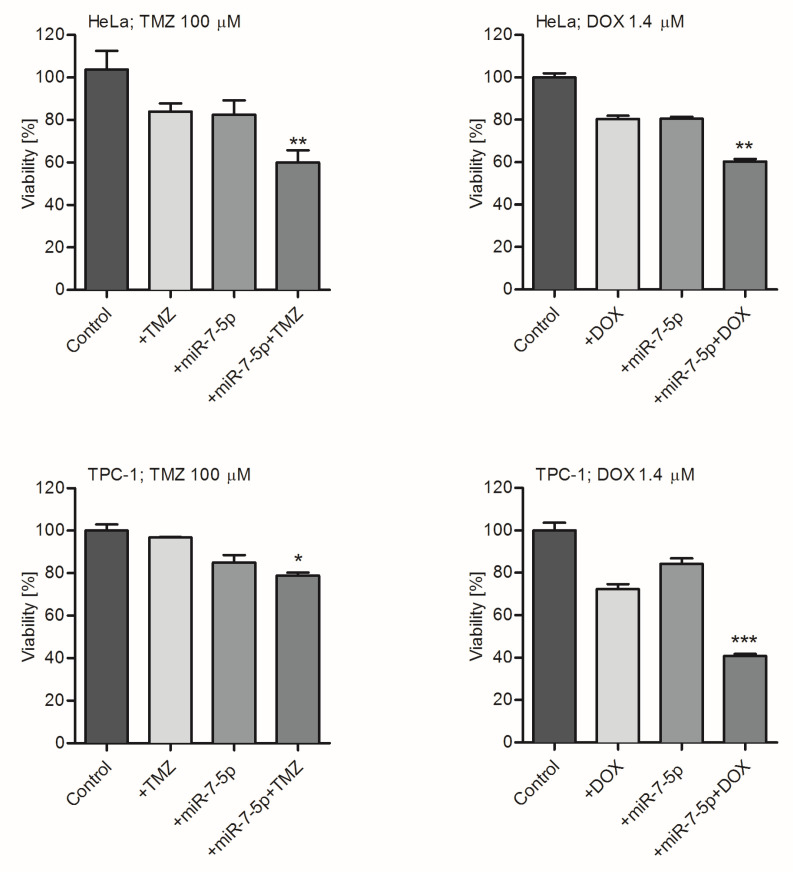
Increased sensitivity of cancer-derived cell lines treated with miR-7-5p to chemotherapeutics is not cell type-specific. Viability analysis (MTS assay) of HeLa (upper panel) and TPC-1 (lower panel) cells treated with miR-7-5p and TMZ (100 µM) or DOX (1.4 µM) for 48 h. Cells treated with miR-NEG served as a control. Data are presented as mean ± SD. * *p* < 0.05; ** *p* < 0.01; *** *p* < 0.001.

Subsequently, it was explored whether the miR-7-5p effect on tumor cell sensitivity is cell/tissue type-specific, and if it can be observed in types of tumor cells other than GB-derived A172 and T98G. To examine this hypothesis, model cell lines derived from cervical cancer (HeLa) and papillary thyroid carcinoma (TPC-1) were treated with miR-7-5p and TMZ (100 µM) or DOX (1.4 µM), as described in the Materials and Methods section. It was found that HeLa cells incubated with TMZ or DOX, in the presence of miR-7-5p, were more susceptible to chemotherapeutic agents (by 40%; Figure 4; upper panel), in comparison to controls. Consequently, the effect of miR-7-5p was observed in TPC-1 cells co-treated with drugs, wherein the viability of cells exposed to TMZ or DOX and miRNA was reduced by 20 and 60%, respectively (Figure 4; lower panel).

**Figure 5:**
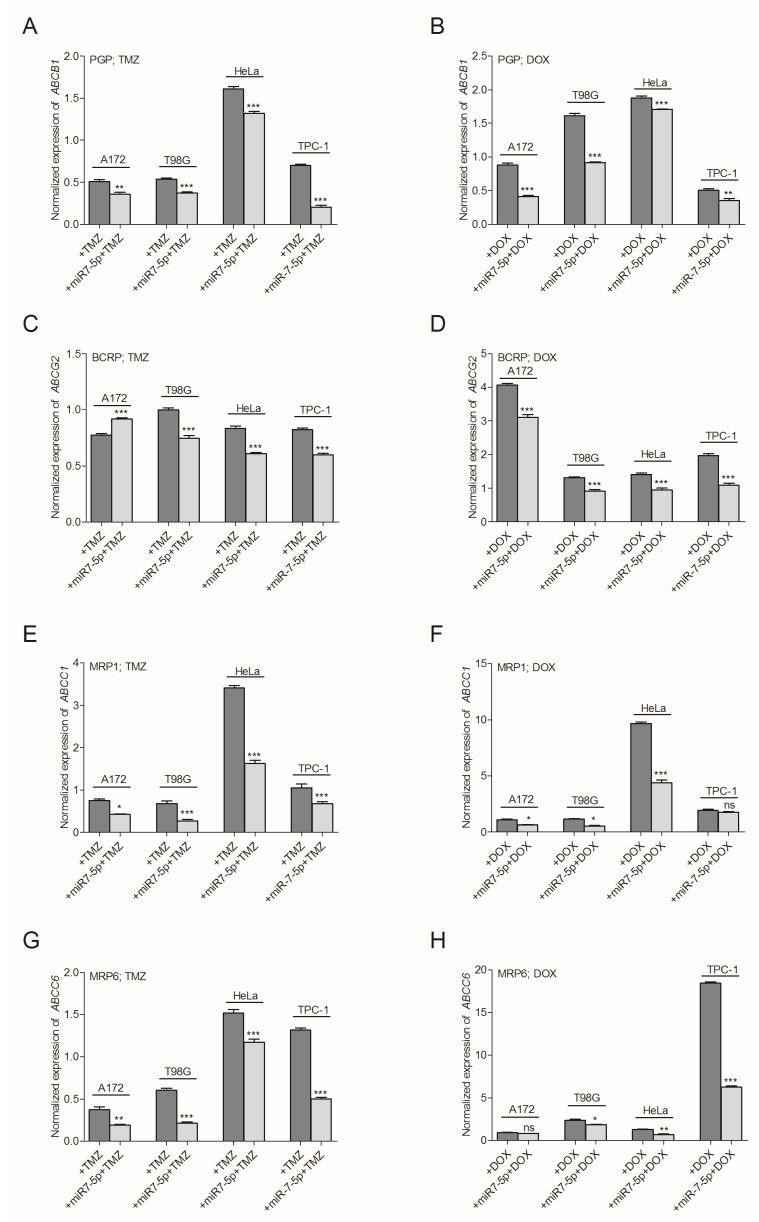
Transfection of A172, T98G, HeLa and TPC-1 cells with miR-7-5p is accompanied by a decrease in the expression of MDR encoding genes. Normalized expression (RT-qPCR) of MDR encoding genes: (**A**,**B**) *ABCB1* gene encoding for PGP, (**C**,**D**) *ABCG2* gene encoding for BCRP, (**E**,**F**) *ABCC1* gene encoding for MRP1, (**G**,**H**) *ABCC6* gene encoding for MRP6 in A172, T98G, HeLa and TPC-1 cell lines treated with miR-7-5p and/or TMZ (200 µM) or DOX (1.4 µM) for 48 h, respectively. Cells treated with miR-NEG served as a control. Data are presented as mean ± SD. * *p* < 0.05; ** *p* < 0.01; *** *p* < 0.001.

Based on the data presented in Figure 2 and Figure 4, we hypothesized that the increased sensitivity of the analyzed cells to the co-administered combination of miRNA and a drug might be a consequence of the decreased expression of MDR genes. An analysis of the *ABCB1*, *ABCG2*, *ABCC1* and *ABCC6* mRNA levels in A172, T98G, HeLa and TPC-1 cells revealed that the transfection of cells with miR-7-5p resulted in a significant reduction of the MDR protein-coding transcripts. This tendency was observed for the vast majority of the analyzed MDR genes in all the tested cell lines. Only the TPC-1 cells co-treated with miRNA and TMZ showed no effect on the expression level of the *ABCC1* gene encoding for the MRP1 efflux pump. Further, the MRP6 transcript was not affected in A172 cells co-treated with miRNA and DOX (Figure 5).

### 2.4. Drug-Loaded Nanocarriers Effectively Kill Tumor Cells in the Presence of miR-7-5p

**Figure 6:**
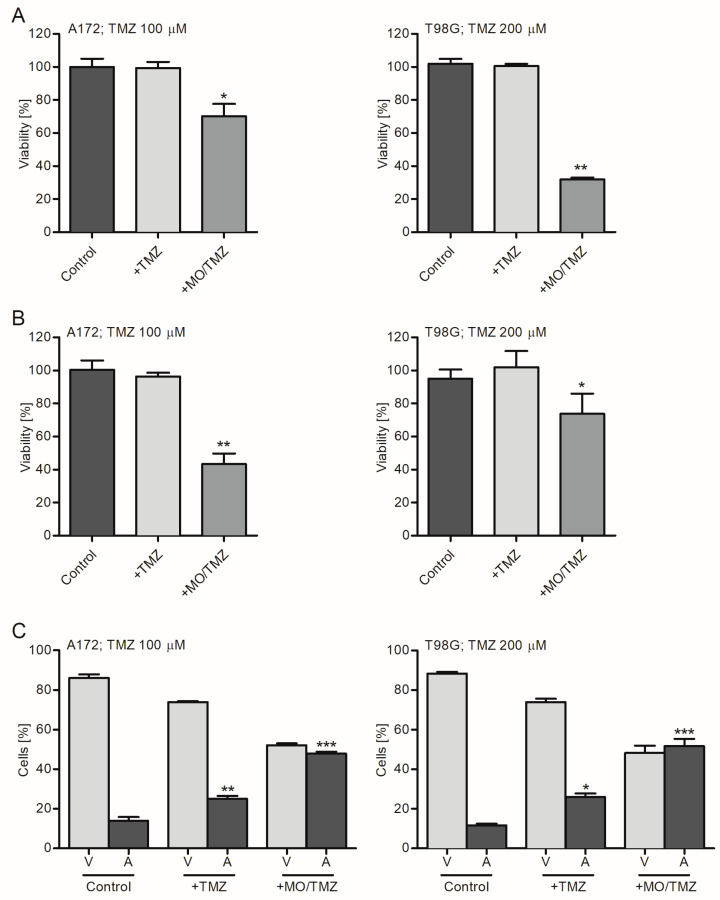
Treatment of A172 and T98G cells with TMZ-loaded MO nanoparticles results in significant reduction of the cells’ viability. Graphs present an analysis of viability (**A**,**B**; MTS and trypan blue exclusion assays, respectively) and apoptosis (**C**; flow cytometry) of A172 and T98G cells treated with TMZ (+TMZ; 100 and 200 µM, respectively) or TMZ-loaded cubosomes (+MO/TMZ) for 48 h. Non-treated cells served as a control. Data are presented as mean ± SD. * *p* < 0.05; ** *p* < 0.01; *** *p* < 0.001; V, viable cells; A, apoptotic and necrotic cells.

In our previous studies [2,3], we investigated the effects of DOX-loaded cubosomes on cancer cells. It has been shown that cubosomes are capable of controlled and targeted delivery of DOX to cancer cells in vitro. Here, we found that a similar effect can be achieved with other classes of chemotherapeutics, such as temozolomide (TMZ). The diffraction patterns of empty MO and TMZ-loaded MO (MO/TMZ) were similar. The cubosomes were separately used for the treatment of A172 and T98G cells. As shown in Figure 6 (A and B; MTS and trypan blue exclusion assays data, respectively), the treatment of GB-derived A172 and T98G cells with TMZ-loaded MO nanoparticles (MO/TMZ; 100 or 200 µM of TMZ, respectively) resulted in the strong reduction of the cells’ viability. It was confirmed that cargo-free phases do not affect the survival rates of A172 and T98G cells (Figure S4). Moreover, flow cytometry analysis revealed that the largest population of apoptotic, and the lowest number of viable cells in both cell lines, was noticed in the subgroup of A172 and T98G cells exposed to cubosomes loaded with the drug (Figure 6C).

**Figure 7:**
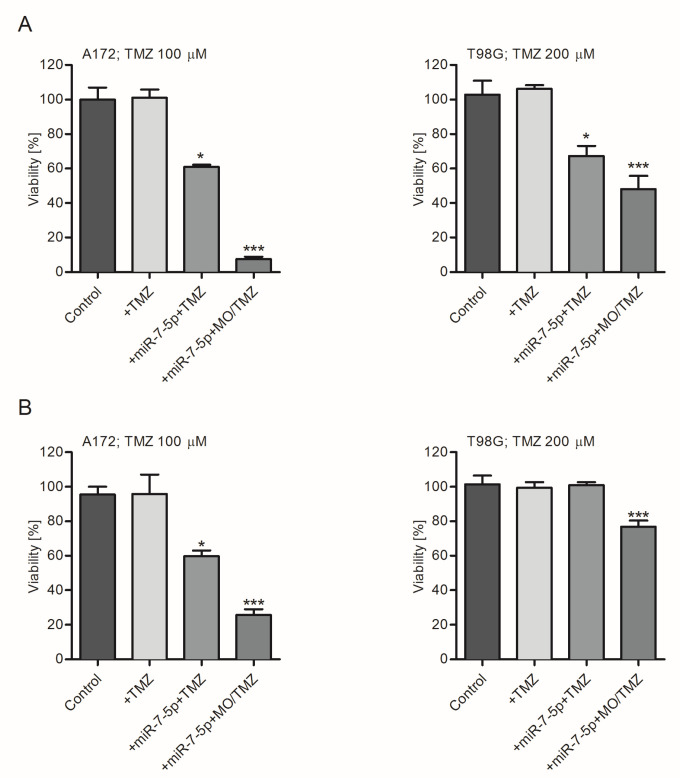
Transfection of A172 and T98G cells with miR-7-5p significantly sensitizes cells to TMZ-loaded cubosomes. Graphs present an analysis of viability (**A**,**B**; MTS and trypan blue exclusion assays, respectively) of A172 and T98G cells exposed to free TMZ (+TMZ; 100 µM and 200 µM, respectively) and miR-7-5p pre-treated cells cultured with free TMZ (+miR-7-5p+TMZ) or TMZ encapsulated within cubosomes (+miR-7-5p+MO/TMZ) for 48 h. Cells treated with miR-NEG served as a control. Data are presented as mean ± SD. * *p* < 0.05; *** *p* < 0.001.

Finally, it was established that the pre-treatment of cells with miR-7-5p, followed by exposure to TMZ-loaded cubosomes, significantly enhances the sensitivity of the treated cells to the chemotherapeutic agent. The strongest reduction in the number of viable cells was observed for miRNA- and MO/TMZ-treated A172 cells (90% drop in the number of viable cells). In the case of drug-resistant T98G cells, around 50% of the cells were affected by consecutive treatments with miR-7-5p- and TMZ-loaded cubosomes (Figure 7).

### 2.5. Drug- and miR-7-5p-Loaded Cubosomes Exhibit Significant Antitumor Properties

**Figure 8:**
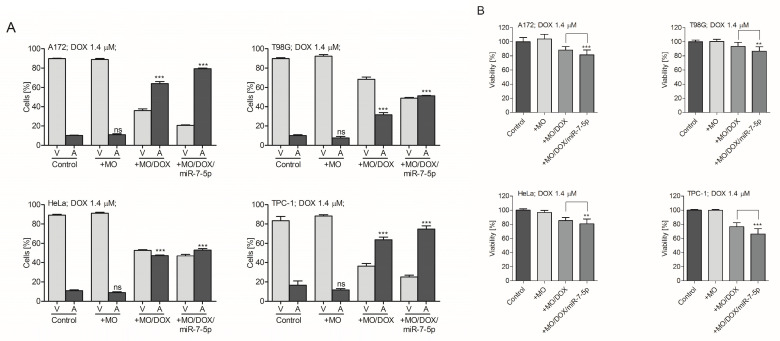
Treatment of A172 and T98G cells with DOX- and miR-7-5p-loaded MO nanoparticles results in significant reduction in the viability of cells. (**A**) Flow cytometry data and (**B**) trypan blue exclusion assays show the percentage of viable and apoptotic or necrotic A172 and T98G cells treated with empty cubosomes (+MO) or DOX-loaded cubosomes (+MO/DOX), or DOX- and miR-7-5p-loaded cubosomes (+MO/DOX/miR-7-5p), for 48 h. Non-treated cells were used as a control. Data are presented as mean ± SD. ** *p* < 0.01; *** *p* < 0.001; ns, non-significant.

As the treatment of A172 and T98G cells with TMZ-loaded MO nanoparticles results in the most significant reduction in the viability of cells pre-transfected with miR-7-5p, it was assessed whether the simultaneous delivery of both (i.e., miRNA and the drug placed in one nanocarrier) can also effectively activate apoptosis in the treated tumor cells. MO cubosomes were simultaneously loaded with DOX (1.4 µM) and miRNA (10 nM). The internal structure of the dual-loaded cubosome formulation was determined using small-angle X-ray scattering (SAXS). Figure S5 shows the diffraction pattern, obtained from the cubosomes loaded with the drug and miR-7-5p (MO/DOX/miR-7-5p). The 1D diffraction pattern presented for the MO-derived cubosomes was a sequence of diffraction peaks with relative positions at ratios of √2, √3, √4, √6 and √8, which can be attributed to the double diamond (Pn3m) symmetry. Overall, the patterns of MO/DOX/miR-7-5p and empty MO or DOX-loaded MO (MO/DOX) were similar [3]. The cubosomes (MO, MO/DOX, MO/TMZ and MO/DOX/miR-7-5p) were separately used for the treatment of A172, T98G, HeLa and TPC-1 cells. After 24 h of co-incubating the cells (separately) with empty, or DOX-loaded or DOX/miR-7-5p, cubosomes, it was observed that the process of apoptosis is most abundant in cells treated with the newly-developed formulation carrying both DOX and miRNA (Figure 8A). The most significant increase in apoptosis was observed in drug-sensitive A172 and TPC-1 cells treated with MO/DOX/miRNA phases. In contrast, the effect of the MO/DOX/miRNA treatment of drug-resistant HeLa and T98G cells was rather modest, but still statistically significant (*p* < 0.05). Trypan blue stain analysis showed that the survival rate pattern in the tested cells overlaps with the flow cytometry data, and that both A172 and TPC-1 cells were mostly affected by the combinatory treatment with drug/miRNA-loaded cubosomes. The impact of MO/DOX/miR-7-5p on the survival of T98G and HeLa cells was also found to be statistically significant (Figure 8B).

**Figure 9:**
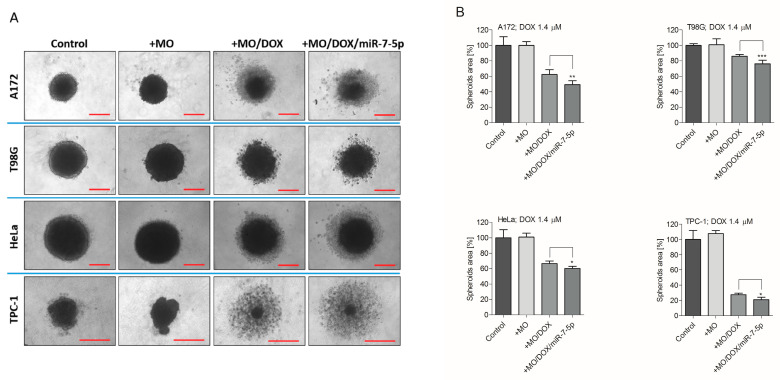
Cubosome-delivered miR-7-5p affects the growth of tumor spheroids. A172, T98G, HeLa and TPC-1 cells grown in a 3D model co-incubated with DOX- and miR-7-5p-loaded cubosomes present significantly reduced dimensions and shape. (**A**) Phase-contrast representative images and (**B**) graphical plots summarizing the average area of spheroids treated with empty cubosomes (+MO) or DOX-loaded cubosomes (+MO/DOX), or DOX- and miR-7-5p-loaded cubosomes (+MO/DOX/miR-7-5p), on day 6. Cells treated with miR-NEG were used as a control. Magnification: 10× lens. Data are presented as mean ± SD. * *p* < 0.05; ** *p* < 0.01; *** *p* < 0.001.

Additionally, to confirm the effect of the nanoparticles on the cells’ viability in vitro, we treated 3D tumor spheroids with empty, DOX-loaded or DOX/miR-7-5p-loaded cubosomes. It was found that the effect of DOX depends on the level of miR-7-5p. Similar to the results of flow cytometry, the growth of spheroids was considerably affected in A172 and TPC-1 cells, while a less significant reduction in the size of the 3D colonies produced by both drug-resistant cell lines (HeLa and T98G) was observed (Figure 9). Collectively, this data indicated that cubosomes co-loaded with the drug and miRNA are capable of effectively suppressing the growth of 3D cellular aggregates.

## 3. Discussion

Nowadays, cancer cases are on the rise, and the available medical therapies are ineffective in reversing the malignant process. An overall improvement in 5-year survival rates has been observed [33], but still, the average rate of mortality is high. Additionally, an important therapeutic barrier is the phenomenon of drug resistance, which is commonly observed in secondary tumors. Therefore, it is necessary to search for new therapeutic methods that would improve the prognosis of cancer patients and increase the effectiveness of existing strategies. The first attempts at novel combinatory treatments considered the usage of siRNA and chemotherapeutics for the treatment of chemo-resistant tumors [34,35]. A significant portion of these attempts primarily focused on the siRNA-mediated knockdown of MDR genes encoding for drug-efflux MRP1 and PGP proteins [36,37,38,39]. One of the currently studied innovative strategies is miRNA-mediated therapy, which can be used alone or in combination with a chemotherapeutic agent distributed to cells using nanocarriers. This idea has been explored over the last decade. Nonetheless, only a few reports have characterized novel bioactive molecules loaded with miRNA and drugs, for application in antitumor treatment. Lee et al. reviewed and listed 18 publications related to the application and therapeutic effects of nanoparticles used for delivering miRNAs and drugs to cancer cells [40]. Nevertheless, most of the studied loaded nano-vehicles were tested against one type of tumor, and there are no data on the universal combinatory treatment tools capable of affecting various types of cancer. Furthermore, until now, only a few reports have directly focused on the treatment of GB, the most frequently diagnosed primary brain tumor in adults, which is especially hard to cure due to its fast development of drug resistance. Bertucci et al. tested the application of anti-miR221 polyarginine-peptide nucleic acid, conjugated with the surface of TMZ-loaded mesoporous silica nanoparticles, and confirmed a strong effect of combinatory nanosystems on the induction of apoptosis in drug-resistant T98G cells [41]. In another study, Costa et al. analyzed intravenously-administered chlorotoxin-coupled nucleic acid lipid particles for the delivery of anti-miR-21 oligonucleotides to GB cells, combined with sunitinib, which decreased the proliferation of tumor cells and tumor size in the murine model [42]. 

Here, we present a study on miR-7-5p and non-lamellar lipids (cubosomes) loaded with miRNA and/or a drug, and the successful use of them to treat various cancer types. Based on our best knowledge, only one study by Cui et al. described the usage of miR-7 and paclitaxel-loaded monomethoxy(poly(ethylene glycol))–poly(d,l-lactide-co-glycolide)–poly(l-lysine), a polymer-based nanoparticle, in the simultaneous co-treatment of ovarian cancer [43]. In contrast, the cubosomes used in our study are lipid-based nanocarriers, and are considered as one of the most promising nanotools. As mentioned before, cubosomes increase the efficiency of transport, and when conjugated with a specific ligand, are capable of targeted drug placement, which results in the intracellular accumulation of the chemotherapeutic agent and provides a faster therapeutic effect [2,3]. Cubic phases consist of a single lipid bilayer, and due to their nature and biochemical properties, they are highly stable and exhibit low toxicity, and therefore are considered as effective drug shuttle systems [44]. Moreover, our recent data (in press) indicate that cubosomes likely fuse with the plasma bilayer membrane, and release cargo in contact with the cellular membrane. Therefore, the delivered cargo escapes the attack of lysosomal enzymes, and thus, the delivered drugs and miRNA are left intact, and may affect the nucleic acids. This mechanism stays in accordance with the published tailor cubosome–cell fusion model [45]. MiR-7-5p, which we examined in our study, has been shown to be significantly downregulated in various human tumors, including glioma, melanoma, breast, papillary and others [22,46,47,48]. We confirmed that the level of miR-7-5p was also significantly downregulated in the cohort of Polish GB specimen’ analyzed in our study. Generally, the role of miR-7-5p is linked with the regulation of cell–cell interactions, adhesion, proliferation, apoptosis and epithelial cell polarity, including its effect on the expression of Bcl-2, Xiap, EGFR and SATB-1, and RAF1 oncogenes [23,26,49]. Moreover, the development of drug sensitivity, which accompanies the decrease in miRNA-7-5p level, might be associated with, among others, the Yin Yang 1 (YY1) protein, which is a transcription factor [46,50]. Nevertheless, the biological importance of the intracellular depletion of miR-7-5p yield in tumor cells is still unclear. Our results have shown that, despite the lack of a significant effect of miRNA-7-5p and TMZ, or DOX alone, on the proliferation and viability of the tested cells, miRNA-7-5p delivery in combination with a drug caused a significant decrease in the survival of the treated cells. We linked this observation with reducedchemoresistance of miR-7-5p-treated cancer cells. We provided a comprehensive analysis of miR-7-5p and MDR transcripts in four functionally different cancer cell lines, as well as GB specimens. The obtained data indicate a direct correlation between the low intracellular level of miR-7-5p and the activation of MDR genes. It was shown that the reconstitution of the miR-7-5p level resulted in the decreased expression of PGP, BCRP, MRP1 and MRP6 encoding genes in GB-derived (A172 and T98G), cervical carcinoma-derived HeLa and PTC-derived TPC-1 cells. These data were supported by analyses performed on a series of GB specimens, which showed the association between a drop in miR-7-5p expression and the upregulation of PGP, MRP1 and MRP6 encoding genes. Importantly, our data overlap with the report on the modulation of oxaliplatin resistance in hepatocellular carcinoma through the miR-7-5p/*ABCC1* axis [51]. Finally, our study also revealed that the function of miR-7-5p is universal, not only in the context of the origin of the tested cell lines. We found that a combinatory treatment with miR-7-5p and any of the tested chemotherapeutic agents (TMZ, DOX or BCNU) led to the sensitization of cancer cells to the administered compound. In conclusion, the available data indicate that miRNA-7-5p plays a crucial role in conducting the cancer cells’ response to drug treatment. Our concept for the potential application of the drug (DOX) and miR-7-5p using cubosomes as a delivery tool was confirmed using a multicellular tumor 3D spheroid model. Therefore, miR-7-5p can be considered as a potential therapeutic compound, which, when used together with a drug, may promote the apoptosis of tumor cells, and as a result, prompt better clinical results and increase the patients’ chances for recovery.

## 4. Materials and Methods

### 4.1. Tissue Samples

Glioblastoma samples were obtained from patients undergoing the surgical resection of tumors at the Medical University of Bialystok in Poland. Immediately after resection, tumor specimens were preserved in 5 volumes of RNAlater Stabilization Solution (Thermo Fisher Scientific; Waltham, MA, USA) and stored overnight at 4 °C in order to allow the reagent to permeate the tissues. Next, the supernatant was discarded and the preserved samples were stored at −80 °C for further processing. All tissue sections (*n* = 23) were histologically evaluated to confirm glioblastoma. Descriptive characteristics of the patients are provided in Table S1 (supplementary materials). The Ethics Committee of the Medical University of Bialystok, Poland approved the study (No. R-I-002/63/2014; 27 February 2014). Written informed consent was obtained from all the patients before surgery.

### 4.2. Cell Cultures

Human glioblastoma-derived A172 (TMZ-sensitive) and T98G (TMZ-resistant) cell lines, as well as the human cervical carcinoma-originated drug-resistant HeLa cell line, were purchased from the American Type Culture Collection (ATCC; Manassas, VA, USA). Dr L. Santoro, from the University of Naples Federico II, Naples, Italy, kindly provided the human papillary thyroid carcinoma-derived TPC-1 cell line. The glioblastoma cell lines were cultivated in high glucose Dulbecco’s Modified Eagle Medium (DMEM; Corning; Corning, NY, USA), supplemented with 10% fetal bovine serum (FBS; HyClone; Pittsburgh, PA, USA) and 1% Antibiotic Antimycotic Solution (Sigma-Aldrich; Steinheim, Germany), while the HeLa and TPC-1 cell lines were grown in RPMI 1640 medium (HyClone; Pittsburgh, PA, USA) supplemented with 10% FBS (HyClone; Pittsburgh, PA, USA) and Antibiotic Antimycotic Solution (Sigma-Aldrich; Steinheim, Germany). Cell cultures were maintained at 37 °C in a humidified, 5% CO_2_ atmosphere.

### 4.3. Preparation of Cubosomes

Monoolein (1-oleoyl-rac-glycerol; purity ≥ 99%), DOX, TMZ and Pluronic F108 (PF108) were used for the synthesis of mesophases. All reagents were purchased from (Sigma-Aldrich; Steinheim, Germany). Solutions were prepared with Milli-Q water (18.2 MΩ/cm^−1^; Millipore; Billerica, MA, USA) with the exception of TMZ, which was dissolved in dimethyl sulfoxide (DMSO; (Sigma-Aldrich; Steinheim, Germany). Cubosomes were prepared according to a slightly modified procedure [2]. Pluronic F108 was selected to provide colloidal stability of formulations and to avoid aggregation of particles. Monoolein and PF108 were first dissolved in chloroform (1 mL) and mixed using a magnetic stirrer until the samples were visually homogeneous. The lipid to polymer ratio was 8:1. Chloroform was subsequently evaporated under a stream of argon (Ar); additionally, for complete solvent removal, the mixtures were placed under vacuum overnight, and then water (1 mL) was added to the lipids and the mixture was homogenized. The concentration of lipids in the resulting dispersions was typically 5% (*w*/*w*). Homogenization was carried out using Ultra-Turrax (IKA; Staufen, Germany) at 16,000 rpm for 40 min. Drug-loaded cubosomes were prepared by mixing DOX (0.45 mg) or TMZ (6 mg) with the melted lipid mixture, and homogenized as described above. TMZ was dissolved in 100 µL of DMSO (Sigma-Aldrich; Steinheim, Germany) prior to mixing with monoolein. To form miR-7-5p-loaded MO carriers (cubosomes), before lipid hydration, miR-7-5p (hsa-miR-7-5p mimic; assay ID: MC1175; Thermo Fisher Scientific; Waltham, MA, USA; 5 nmol) was dissolved in 50 µL of Milli-Q water, and subsequently this solution was added to the lipid mixture to form the cubic phase, left for 4 h and then processed as described above.

### 4.4. Small Angle X-Ray Scattering (SAXS)

Measurements were performed using a Bruker Nanostar system working with CuKα radiation, equipped with a Vantec 2000 area detector. The samples were loaded into 1.5-mm capillaries before measurement, and the scattered intensity was collected over 2 h. Measurements were performed at 25 °C. The scattering vector (q) was determined from the scattering angle using the relationship q = (4π/λ)sinθ, where 2θ is the scattering angle and λ is the wavelength of radiation. To identify the phase type, the scattering vector (q) values of the peaks were correlated with the Miller indices for known mesophases.

### 4.5. MiRNA Transfection and Treatment of Cells with Chemotherapeutics

Cells were transfected with 40 nM of miR-7-5p or miR-negative (control; miR-NEG; #4466058; Thermo Fisher Scientific; Waltham, MA, USA) using Lipofectamine 3000 reagent (Thermo Fisher Scientific; Waltham, MA, USA), according to the manufacturer’s instructions. After 24 h of incubation, the medium was supplemented with TMZ dissolved in DMSO (Sigma-Aldrich; Steinheim, Germany) at a final concentration of 100 µM for A172, HeLa and TPC-1, and 200 µM for T98G. When required, nanoparticles (MO), empty or loaded with TMZ, were added to the media: 3.2 or 6.4 µL/mL, to achieve the TMZ concentrations of 100 and 200 µM, respectively. Optionally, cell lines were also treated with DOX at a final concentration of 1.4 µM or carmustine (BCNU; 2.5–25 µM; TargetMol; Wellesley Hills, MA, USA) or nanoparticles (1.8 µL/mL) loaded with DOX, or simultaneously DOX and miR-7-5p, at concentrations of 1.4 µM and 10 nM, respectively. The cells treated with only miR-NEG served as a control.

### 4.6. RNA Extraction, Quality Assessment and Quantitative Real-Time PCR (RT-qPCR)

Total RNA was isolated from the collected glioblastoma specimens using the MirVana Isolation Kit (Thermo Fisher Scientific; Waltham, MA, USA), according to the manufacturer’s protocol. RNA concentration and purity was evaluated by Nano Drop 2000/200c (Thermo Fisher Scientific; Waltham, MA, USA), whereas the integrity of samples was checked using the Agilent RNA 600 Nano Kit (Agilent Technologies; Winooski, VT, USA) and Bioanalyzer 2100 (Agilent Technologies; Winooski, VT, USA). Only samples with RIN (RNA Integrity Number) values of 6 or greater were taken for further processing. The expression of miR-7-5p was quantified via the RT-qPCR method using TaqMan assay (Thermo Fisher Scientific; Waltham, MA, USA) for miRNA, according to the manufacturer’s protocol. Briefly, cDNA was generated with specific primers dedicated to miR-7-5p (assay ID: 000268) and RNU6B (assay ID: 001093; endogenous control) and High-Capacity cDNA Reverse Transcription Kit with RNase Inhibitor (Thermo Fisher Scientific; Waltham, MA, USA) followed by TaqMan-based RT-qPCR. Reactions were performed with specific primers and probes (Thermo Fisher Scientific; Waltham, MA, USA), TaqMan Universal Master Mix II (Thermo Fisher Scientific; Waltham, MA, USA) and cDNA under conditions recommended by the manufacturer. Amplification, data acquisition and analyses were carried out using the Applied Biosystems 7900HT Fast Real-Time PCR System (Thermo Fisher Scientific; Waltham, MA, USA). Commercial FirstChoice Human Brain Reference RNA (Thermo Fisher Scientific; Waltham, MA, USA) served as a non-tumor control.

Total RNA from the cell lines was isolated using the GeneMATRIX Universal RNA Purification Kit (EURx; Gdansk, Poland), following the producer’s instructions. Next, 500 ng of RNA were converted to single-stranded cDNA using High-Capacity cDNA Reverse Transcription Kit with RNase Inhibitor (Thermo Fisher Scientific; Waltham, MA, USA). The gene expression of the patients’ samples and treated cell lines was performed with Maxima SYBR Green/ROX qPCR Master Mix (2X; Thermo Fisher Scientific; Waltham, MA, USA). RT-qPCR was performed using the CFX96 Detection System (Bio-Rad; Hercules, CA, USA) under the following conditions: 95 °C/30 s, 40 cycles at 95 °C/5 s, 58 °C/15 s and 72 °C/10 s. The primers (0.4 µM) used in the assay are listed in Table 1. Data were analyzed using Bio-Rad CFX Manager software (version 3.1, Bio-Rad; Hercules, CA, USA). Relative fold changes in gene expression were calculated using the comparative 2^ΔΔCt^ method and normalized to the corresponding reference genes. Commercial FirstChoice Human Brain Reference RNA was used as a non-tumor control.

### 4.7. MTS Test

Viability of miRNA-treated cells exposed to the drug or drug-loaded nanoparticles was evaluated by an MTS-based test (CellTiter 96 AQueous One Solution Cell Proliferation MTS Assay; Promega; Madison, WI, USA). The transfection mixture containing miR-7-5p or miR-NEG was added to the wells of a 96-well plate. Next, cells suspended in 80 µL of complete medium (1.2 × 10^3^ per well) were applied to the wells and incubated overnight. The next day, media were supplemented with empty nanoparticles, drug-loaded nanoparticles or TMZ at a final concentration of 100 µM for A172, HeLa or TPC-1, and 200 µM for the T98G cell line. Alternatively, cell lines were treated with DOX (1.4 µM) or BCNU (2.5–25 µM). After 48 h, MTS reagent (20 µL per well) was added to each well and cells were incubated for another 3 h. Absorbance was measured at 490 and 650 nm in a microplate reader (Synergy 2; BioTek Instruments; Winooski, VT, USA). Results were expressed as the percentage of proliferating cells compared to the untreated controls.

### 4.8. Trypan Blue Dye Exclusion Assay

Trypan blue assay was used to assess the number of viable cells compared to the total quantity of cells. The experiment was performed as previously described, with minor modifications [2]. Briefly, after placing the transfection mixture containing miR-7-5p or miR-NEG into the 12-well plate, cells suspended in 1 mL of complete medium (1 × 10^5^ cells) were added. The next day, the media were supplemented with empty/drug-loaded nanoparticles and free drugs in concentrations suitable for the cell lines, as described above. After 48 h, all cells (both attached and unattached) were harvested, pelleted (200× *g*, 5 min), resuspended in Dulbecco’s phosphate buffered saline (D-PBS; HyClone; Pittsburgh, PA, USA) and stained for 10 min with trypan blue (NanoEnTek Inc.; Seoul, Korea) at a final concentration of 0.2%. The number of total and viable cells was determined via an automatic EVE cell counter (NanoEnTek Inc.; Seoul, Korea). Results are expressed as the percentage of viable cells.

### 4.9. Annexin V-Based Apoptosis Assay

The percentage of viable and apoptotic cells was determined using flow cytometry, as described previously with minor changes [52]. Cells were treated, as described above, in the trypan blue exclusion assay section. After 48 h of exposure to free drug or drug- and miR-7-5p-loaded nanoparticles, all the cells were harvested, washed with D-PBS once and resuspended in 500 µL of Annexin V Binding Buffer (BD Biosciences; San Jose, CA, USA) followed by staining with Annexin V conjugated with fluorescein isothiocyanate (Annexin V-FITC; 5 µL; BD Biosciences; San Jose, CA, USA) for 15 min in the dark. Next, the samples were examined using the BD Accuri C6 Plus flow cytometer and dedicated BD Biosciences software (version 1.0.23.1; BD Biosciences; San Jose, CA, USA). Data are presented as the percentage of viable and apoptotic cells.

### 4.10. Spheroids Formation Assay

Cell lines were seeded on ultra-low attachment 96-well round-bottom plates (Corning; Corning, NY, USA) at 5000 cells per well. After 24 h, the formed spheroids were treated with empty nanoparticles or nanoparticles loaded with DOX, or DOX and miR-7-5p. Spheroids were cultured with nanoparticles for 6 days and pictures were captured using Observer D1 microscope (Zeiss; Oberkochen, Germany; 10× lens) equipped with Axio Vison LE software (Zeiss; Oberkochen, Germany). The area of spheroids was calculated using ImageJ tool (NIH; Bethesda, MD, USA). The results are presented as the percentage of spheroid area compared to controls.

### 4.11. Data Analysis

All the obtained results were analyzed using Prism software (GraphPad; San Diego, CA, USA). For statistical purposes, one-way ANOVA followed by Bonferroni post-hoc comparative tests were used. Statistical significance was considered at *p* < 0.05. Data are presented as mean ± SD. Figures were created using Prism software (version 5.04, GraphPad; San Diego, CA, USA). All experiments were performed at least in triplicates.

## Figures and Tables

**Table 1:** List of primers used in the studies.

Gene Name	Nucleotide Sequences (5′ → 3′)
*ABCC1*	F: TGTGGGAAAACACATCTTTGA
	R: CTGTGCGTGACCAAGATCC
*ABCC6*	F: TGTCGCTCTTTGGAAAATCC
	R: AGGAACACTGCGAAGCTCAT
*ABCG2*	F: GGTGGAGGCAAATCTTCGTTATTAGA
	R: GAGTGCCCATCACAACATCATCTT
*ABCB1*	F: CAGGAACCTGTATTGTTTGCCACCAC
	R: TGCTTCTGCCCACCACTCAACTG
*MGMT*	F: CCTGGCTGAATGCCTATTTC
	R: GATGAGGATGGGGACAGGATT
*18S rRNA* (endogenous control for cell lines)	F: CCAGTAAGTGCGGGTCATAAG
	R: CCATCCAATCGGTAGTAGCG
*TBP* (endogenous control for patients’ specimens)	F: GAGCTGTGATGTGAAGTTTCC
	R: TCTGGGTTTGATCATTCTGTAG

## Supplementary Materials

Supplementary materials can be found at https://www.mdpi.com/1422-0067/21/14/5039/s1. Table S1. Descriptive characteristics of the GB patients. Figure S1. Comprehensive evaluation of expression of miR-7-5p and MDR genes in GB specimens (*n* = 23) determined by RT-qPCR. Figure S2. The intracellular level of miR-7-5p is strongly enhanced in the A172 cells transfected with miR-7-5p for 48 h. Cells treated with miRNA negative (miR-NEG) served as a control. The data were determined using RT-qPCR and are presented as mean ± SD. *** *p* < 0.001. Figure S3. Drug-resistant T98G cells present enhanced sensitivity to BCNU (5 µM) when pre-treated with miR-7-5p. Viability analysis (MTS assay) of the T98G cell line treated with miR-NEG (left panel) or miR-7-5p (right panel) and various concentrations of BCNU (2.5–25 µM) for 48 h. Data are presented as mean ± SD. * *p* < 0.05; ** *p* < 0.01. Figure S4. Drug-free cubosomes do not affect the viability of A172 and T98G cells. Graphs present an analysis of the viability (A, B; MTS and trypan blue exclusion assays, respectively) of A172 and T98G cells treated with TMZ (+TMZ; 100 and 200 µM, respectively) or cargo-free cubosomes (+MO) for 48 h. Non-treated cells served as a control. Data are presented as mean ± SD. Figure S5. The structure of cubosomes is not affected by miR-7-5p and DOX. The diffraction pattern of the newly developed miR-7-5p- and DOX-loaded cubosomes (MO/DOX/miR-7-5p) was determined by small angle X-ray scattering (SAXS).

## Abbreviations

ATCC	American Type Culture Collection
BCNU	carmustine
BCRP	breast cancer resistance protein
DMEM	Dulbecco’s Modified Eagle Medium
DMSO	dimethyl sulfoxide
DOX	doxorubicin
D-PBS	Dulbecco’s phosphate buffered saline
FBS	fetal bovine serum
FITC	fluorescein isothiocyanate
GB	glioblastoma
MDR	multidrug resistance
miR-7-5p	hsa-miR-7-5p mimic
miR-NEG	miRNA negative control
miRNA /miR	microRNA
MO	monoolein cubosomes
MRP1	multidrug resistance-associated protein 1
MRP6	multidrug resistance-associated protein 6
MGMT	O^6^-methylguanine methyltransferase
PGP	P-glycoprotein
RIN	RNA Integrity Number
RT-qPCR	quantitative real-time PCR
SAXS	small angle X-ray scattering
TMZ	temozolomide
