# Supplementary material for: Combinatory Treatment with miR-7-5p and Drug-Loaded Cubosomes Effectively Impairs Cancer Cells
Source: Int J Mol Sci. 2020 Jul 17;21(14):5039. doi: 10.3390/ijms21145039 (PMC7404280; doi:10.3390/ijms21145039)
Supplement: Supplementary file 1 [file ijms-21-05039-s001.zip › Table S1.pdf]

Table S1. Descriptive characteristics of the GB patients (n=23).

**Characteristic**	**GB patients**		
**Age (years)**	Gender, n (%)		Total, n (%)
	Female	Male	
**<40**	2	1	3 (13)
**40-50**	2	3	5 (22)
**50-60**	1	4	5 (22)
**>60**	5	5	10 (43)
**Total**	10 (43)	13 (57)	23 (100)
**Age (years)**			
**Mean ±SD**	57 ±14		
**Median (min., max.)**	59 (32,78)		
**Localization of GB**	Patient numbers, n (%)		
**Frontal lobe**	10 (43)		
**Temporal lobe**	9 (40)		
**Parietal lobe**	3 (13)		
**Occipital lobe**	1 (4)		
	n (%)	Mean ±SD	Median (min., max.)
**Primary glioblastoma**	19 (82)	61 ±12	65 (33,78)
**Secondary glioblastoma**	4 (17)	42 ±13	38 (32,60)
