# Supplementary figures and images for: Combinatory Treatment with miR-7-5p and Drug-Loaded Cubosomes Effectively Impairs Cancer Cells
Source: Int J Mol Sci. 2020 Jul 17;21(14):5039. doi: 10.3390/ijms21145039 (PMC7404280; doi:10.3390/ijms21145039)

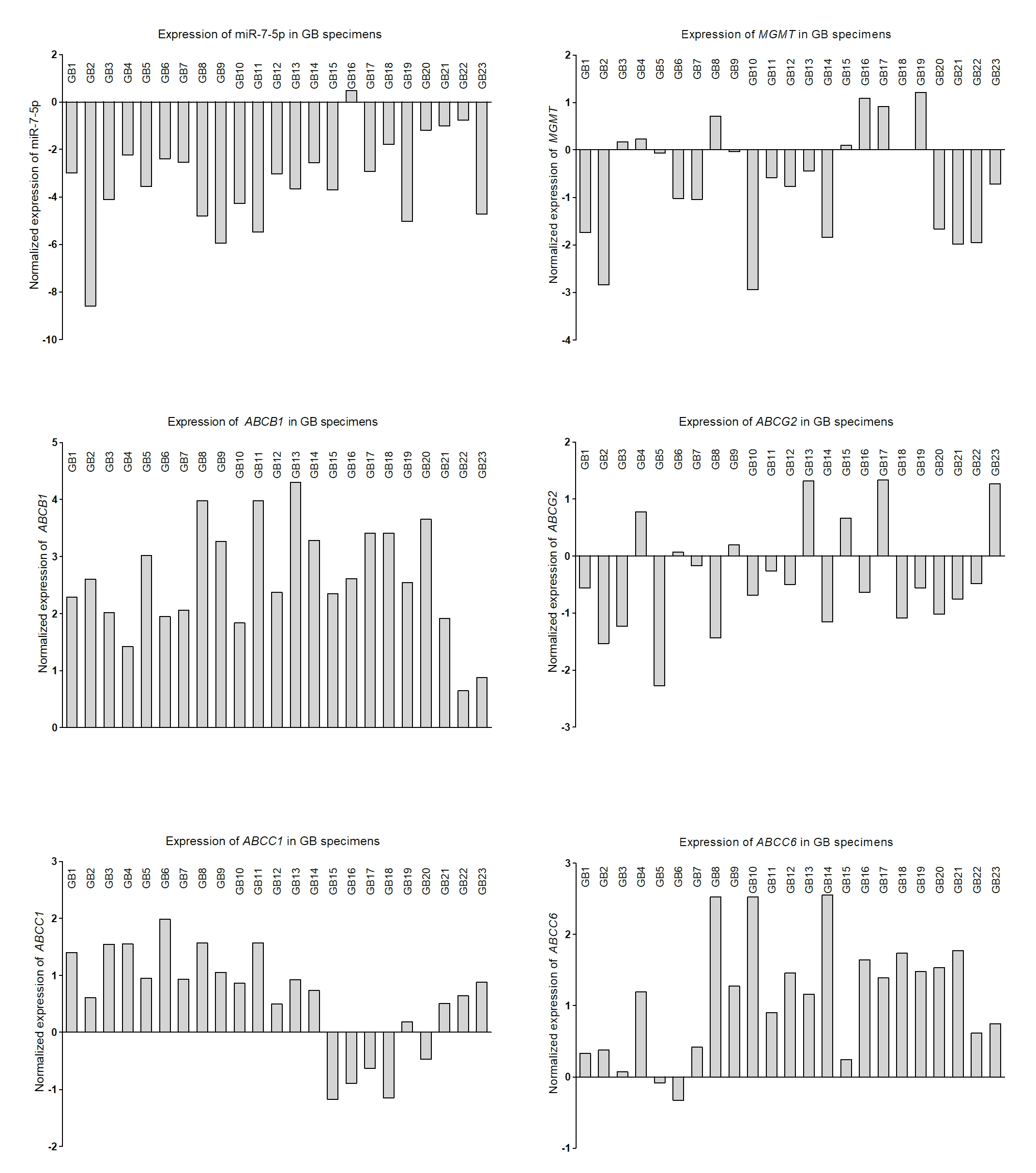

Supplement: Supplementary file 1 [file ijms-21-05039-s001.zip › Figure S1.tif]

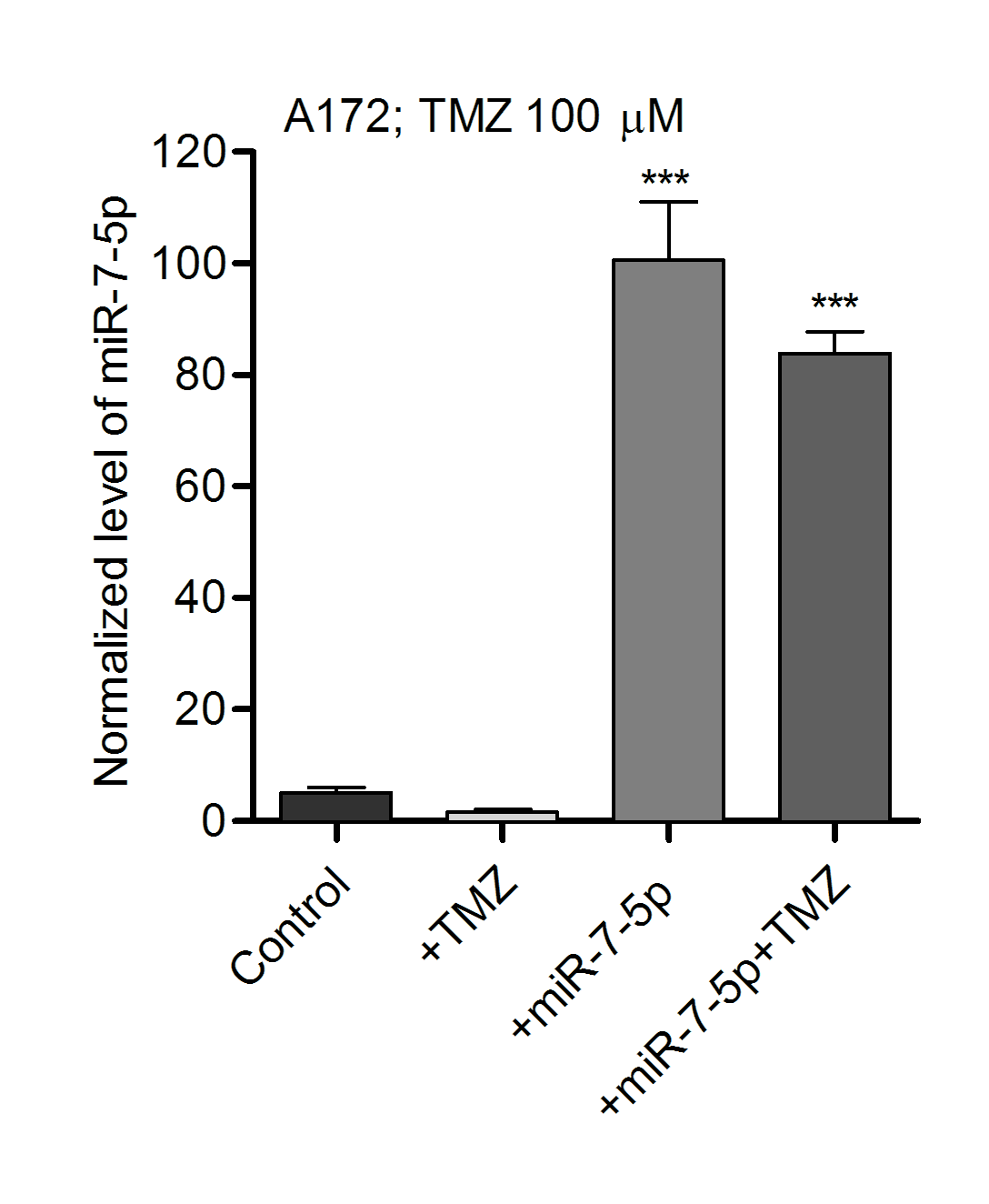

Supplement: Supplementary file 1 [file ijms-21-05039-s001.zip › Figure S2.tif]

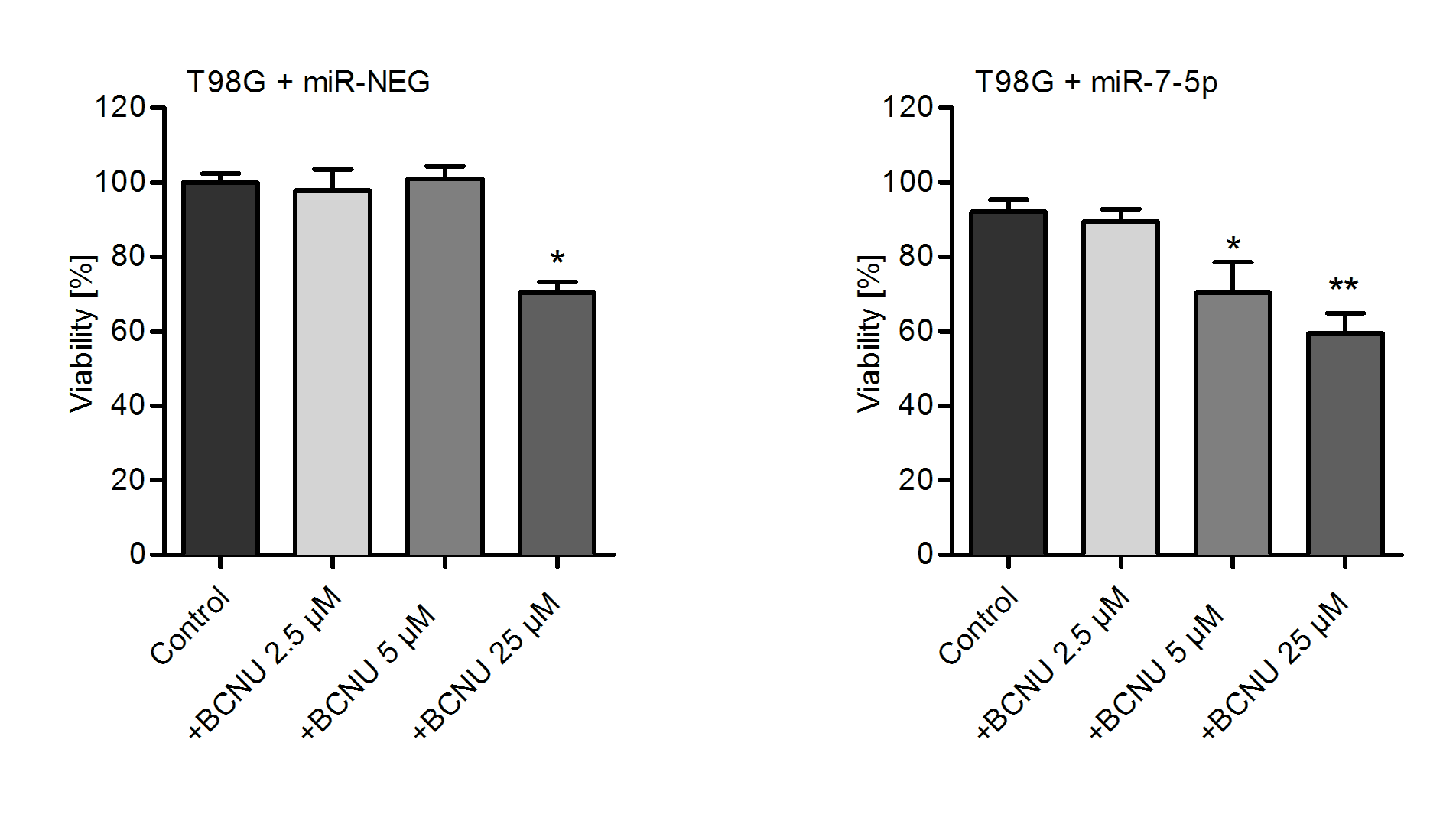

Supplement: Supplementary file 1 [file ijms-21-05039-s001.zip › Figure S3.tif]

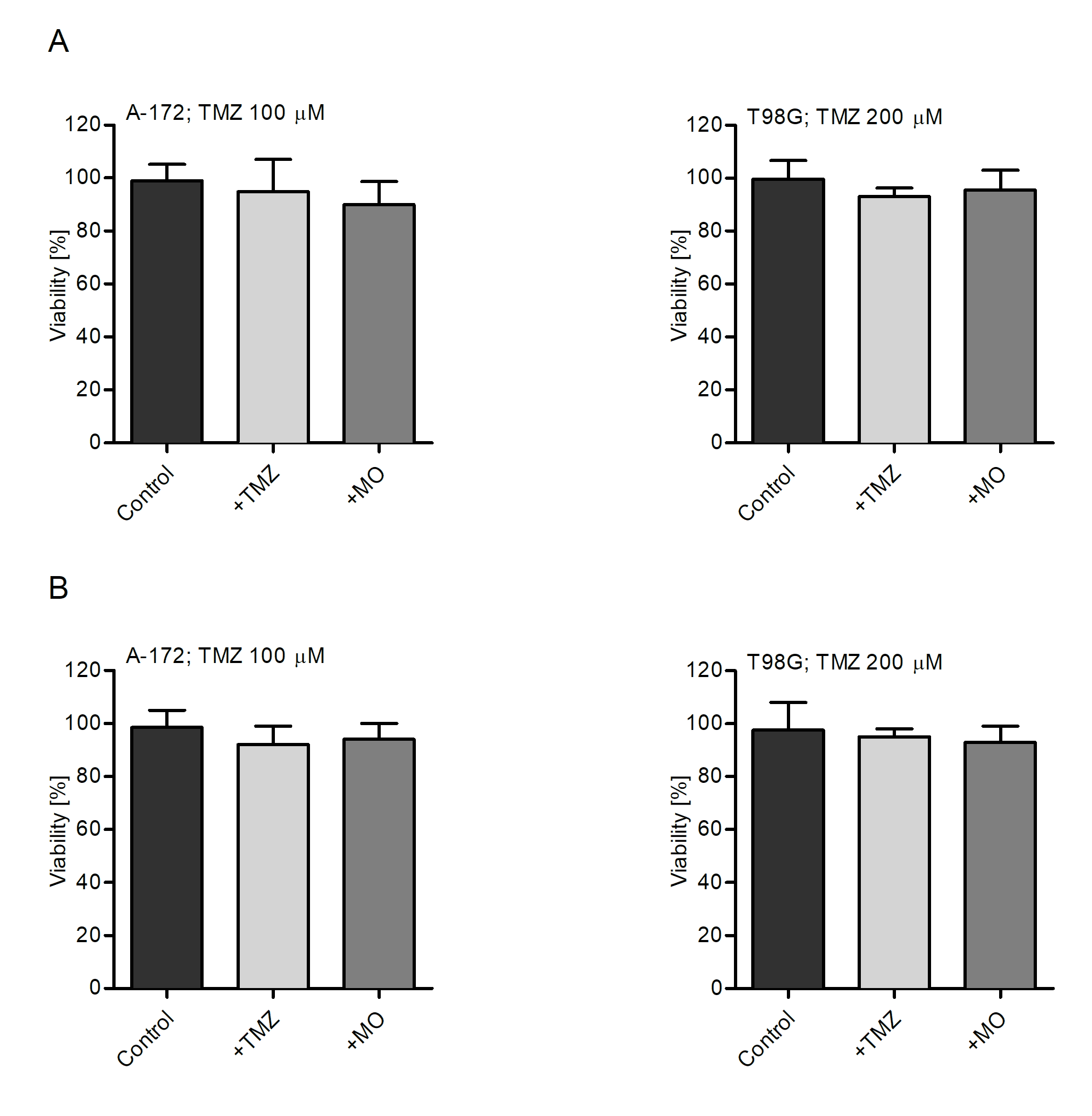

Supplement: Supplementary file 1 [file ijms-21-05039-s001.zip › Figure S4.tif]

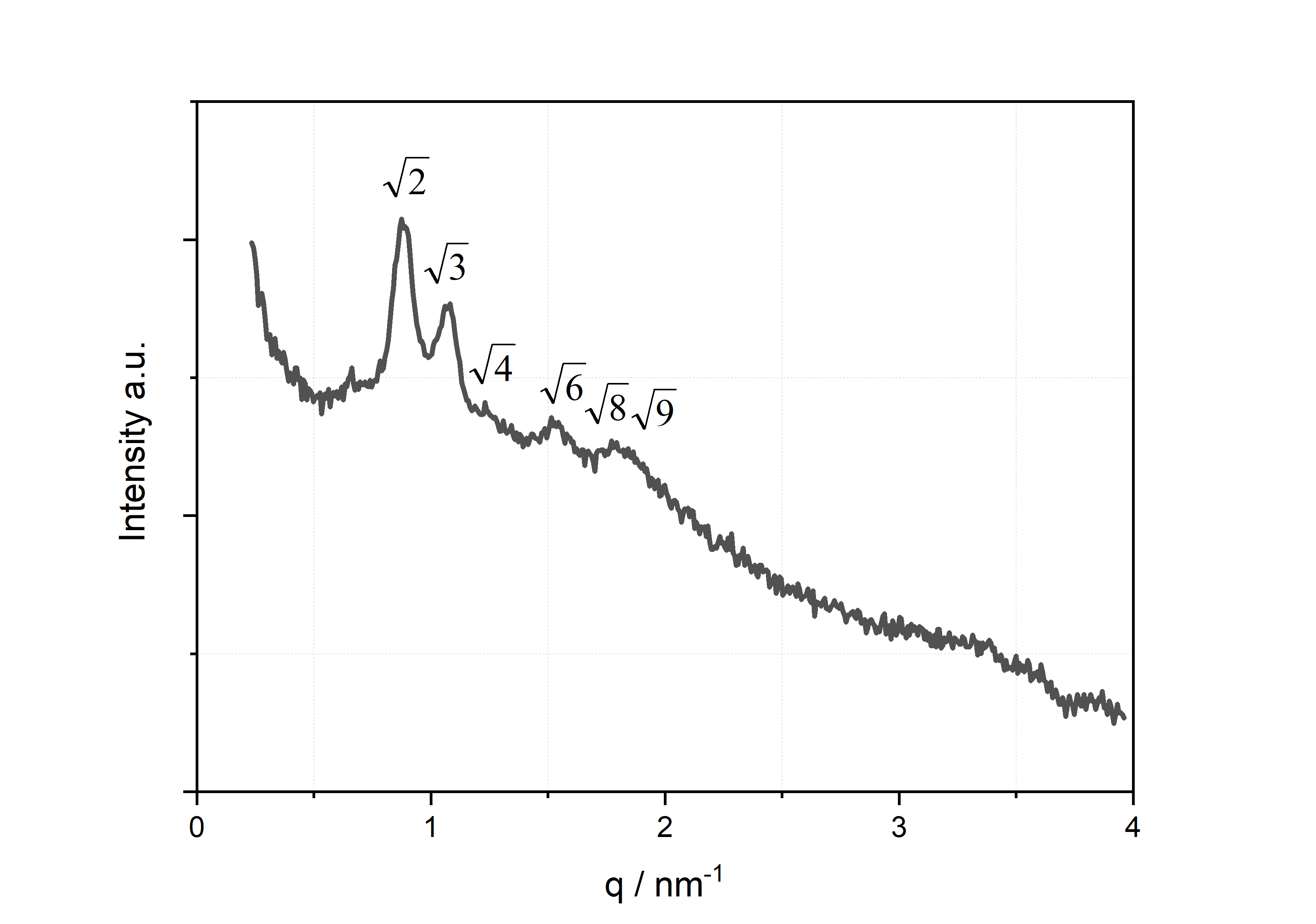

Supplement: Supplementary file 1 [file ijms-21-05039-s001.zip › Figure S5.tif]
